# Exploring the readiness of senior doctors and nurses to assess and address patients’ social needs in the hospital setting

**DOI:** 10.1186/s12913-022-07642-x

**Published:** 2022-02-24

**Authors:** Katherine J. Lake, Mark A. Boyd, Lisa Smithers, Natasha J. Howard, Anna P. Dawson

**Affiliations:** 1 School of Public Health, The University of Adelaide, Adelaide, South 5005 Australia; 2 Adelaide Medical School, Faculty of Health and Medical Sciences, The University of Adelaide, Adelaide, South Australia 5005 Australia; 3 Northern Adelaide Local Health Network, Adelaide, South Australia 5000 Australia; 4 School of Health and Society, The University of Wollongong, Wollongong, New South Wales 2522 Australia; 5 Wardliparingga Aboriginal Health Equity, South Australian Health and Medical Research Institute, Adelaide, South 5000 Australia

**Keywords:** Social Determinants of Health, Social Needs, Healthcare Workers, Australia, In-depth Interviews, Grounded Theory

## Abstract

**Background:**

Adverse social circumstances are a key factor in health outcomes. Hospitals are an opportune setting for assessing and addressing the unmet social needs of patients, however, the readiness of healthcare workers in hospitals to undertake such tasks requires further exploration in the Australian context. This study aimed to generate a theory of doctors’ and nurses’ readiness to assess and address patients’ social needs in a hospital setting.

**Methods:**

A constructivist grounded theory methodology was applied, with purposive and theoretical sampling used to gather diverse perspectives of readiness during semi-structured interviews with twenty senior doctors and nurses from a variety of clinical specialties working in hospitals serving communities experiencing inequitable social and health outcomes. Line-by-line coding, memo writing, and diagramming were used in analysis to construct an interpretive theory of readiness. Application of constant comparison analytic processes were used to test the robustness of the theory.

**Results:**

The readiness of doctors and nurses varies across individuals and departments, and is founded upon a state of being comfortable and confident to assess social need as determined by a range of personal attributes (e.g. knowledge of social need; skills to assess social need); a state of being willing and prepared to assess and address social need facilitated by supportive environments (e.g. departmental culture); and enabling characteristics of the clinical encounter (e.g. time, rapport).

**Conclusions:**

We found that the readiness of doctors and nurses is dynamic and impacted by a complex interplay of personal attributes along with contextual and situational factors. These findings indicate that any efforts to strengthen the readiness of doctors and nurses to assess and address social needs must target personal capabilities in addition to characteristics of the working environment.

## Background

The social determinants of health (SDH) are the “conditions in which people are born, grow, work, live and age” [1]. There is a well-established, evidence-based link between social circumstances and health outcomes. Not only are these linked, they invariably follow a social gradient in that the lower an individual’s socioeconomic position the more likely they are to experience ill health and earlier mortality [2]. ‘Social needs’ are the basic resources that individuals require to live a healthy life, and include personal safety, food security, financial security, safe housing, social connection, and transportation [2, 3]. An absence or insufficiency of basic social needs is associated with poor health outcomes as well as difficulties in healthcare access and utilisation [3]. Unmet social needs can also complicate clinicians’ ability to provide appropriate healthcare [3–5]. In Australia, males and females from the lowest socioeconomic group live on average 5.7 and 3.3 years less, respectively, than individuals from the highest socioeconomic group [4]. To improve the health of communities experiencing disparate health outcomes, more needs to be done to address unmet social needs.

The link between social needs, health outcomes and related burden of disease has led to calls for healthcare services, at all levels, to increase efforts to intervene on patients’ social needs in order to improve both individual and population level health outcomes [6–8]. From a systems perspective, hospitals are integral to the discussion of potential interventions for addressing social needs, as they are one of the few organisations that provide healthcare that is accessible at all times [9, 10]. Research from the United States shows that individuals from a lower socioeconomic position seek acute hospital care over community or outpatient services since it is more accessible and less expensive [11]. In Australia, 24% of emergency department presentations in 2017–2018 were people living in the lowest socioeconomic areas compared to 14% for people living in the highest socioeconomic areas [12]. While hospitals are designed for tertiary healthcare, they clearly play a significant role in the prevention, care, and treatment of individuals whose health needs are caused or exacerbated by one or more unmet social needs [5]. To ensure that hospitals can effectively meet the needs of their patients, any determining factors, barriers, and enablers that impact the ability of clinicians to effectively address social needs in clinical practice must be identified and understood. Evidence regarding behaviours can inform the development of appropriate interventions.

Currently, an understanding of the readiness of healthcare workers (HCW) to identify (assess) and take action on (address) patients’ unmet social needs in the hospital setting is lacking. Previous studies have focused on the readiness of doctors and nurses to use social needs screening tools [3, 13–19], primarily screening tools for domestic violence [13–15, 18, 20–25]. A recent systematic review by Quiñones-Rivera and colleagues [19] found that providers held generally positive attitudes and beliefs about the importance of addressing social needs, however, they identified barriers to social needs screening including concerns regarding patient discomfort at being questioned, increasing pressure on time and workflow, risk to patient-provider relationships, and a lack of knowledge about how to address identified social needs [19]. A number of studies focused on the readiness of doctors and nurses in primary healthcare settings [3, 17, 26–28]. One study identified disparities in screening rates between healthcare providers in the outpatient and inpatient settings, finding that a greater proportion of paediatric residents screened for social needs in the outpatient setting compared to those in the inpatient setting [28].Interestingly, paediatric residents cited the same barriers (time constraints, lack of knowledge, perceived patient discomfort) in both outpatient and inpatient settings [28].

Constructs previously explored relating to clinician readiness to assess and address patients’ social needs include understanding, attitudes, beliefs, perceptions, experience, and knowledge [3, 13–15, 17, 18, 20–22, 24–27, 29]. An understanding of the readiness of doctors and nurses in the hospital context is needed to guide future quality improvement initiatives that focus on workforce and systems responses to unmet social needs associated with adverse health outcomes. The objective of this qualitative study, therefore, was to explore and conceptualise the readiness of doctors and nurses to assess and address patients’ social needs in the hospital setting and to construct a theory of readiness that considered any potential barriers or enablers to readiness.

## Method

### Study Design

We applied constructivist grounded theory to build an interpretive theory of readiness in the context of doctors and nurses assessing and addressing patients’ social needs in a hospital setting [30]. Constructivist grounded theory builds on a pragmatist perspective which situates the research in the contexts, lives, roles and relationships of participants and emphasises the meaning of participant experiences [31, 32]. Constructivist grounded theory uses an interpretive approach that assumes fluid and indeterminate reality, defines multiple perspectives, and studies peoples actions to solve emergent problems [30].

### Context

The project was conducted at the two hospitals (Lyell McEwin Hospital and Modbury Hospital) of the Northern Adelaide Local Health Network (NALHN) in Adelaide, South Australia. These hospitals provide a comprehensive range of services including medical, surgical, emergency and outpatient services to over 400,000 people living in Adelaide’s north and north-eastern suburbs [33], where there is significant social and health inequalities [34, 35]. NALHN has been identified as a hot spot for potentially preventable hospital admissions which is seen to reflect a higher prevalence of acute and chronic conditions and poorer functioning of the non-hospital health care system [36]. NALHN is therefore an opportune setting for social needs interventions within the hospital system that aim to improve the overall health and wellbeing outcomes of individuals and communities in northern Adelaide. Relevant jurisdictional ethical approvals were sought to undertake the research.

### Recruitment Strategies

Purposive and theoretical sampling was used to recruit senior doctors and nurses across NALHN. The heads of each hospital division (Divisional Directors) were first contacted via email by clinical researcher MB, who is employed by NALHN and has existing collegial relationships with the medical and nursing workforce. This email invited the participation of senior Medical and Nursing personnel from each of the NALHN hospital departments and sought advice regarding key informants in a position to explore our research questions. These individuals were then contacted via email with information about the study and an invitation to participate. Those who volunteered to participate were provided with a written information sheet and a verbal description of the study; and any questions were answered prior to seeking informed consent to participate.

### Data Collection

Data for our study was collected in the form of semi-structured interviews [30, 37]. The interview schedule explored constructs of readiness previously described in the literature: understanding, attitudes, beliefs, perceptions, experience, and knowledge [3, 13–15, 17, 18, 20–22, 24–27, 29]. It also explored relevance to clinical practice, comfort and confidence to assess and address unmet social needs, as well as doctors’ and nurses’ perceptions of their own (or others) capacity to assess and address these needs. Additional questions explored what interviewees believed ‘readiness’ to be. We asked participants about both assessing and addressing social needs as we perceived them to be closely linked and we wanted to explore the relationship between the two processes. Given that interviewees were senior doctors and nurses who held managerial and administrative positions as well as clinical roles, they were encouraged to share their own experiences as well as their perceptions of the experiences of junior personnel within their division. In this way, primary data and shadowed data were collected [38].

In total 20 semi-structured interviews were conducted with senior doctors and nurses (11 doctors, 9 nurses, 7 males, 13 females) with a mean age of 49.3 years (range: 24–68 years, two did not disclose). Participants were from a variety of specialties across the NALHN hospitals (e.g., general medicine, emergency medicine, paediatrics) and fulfilled both senior clinical and managerial roles. They reported a mean of 9.7 years working in NALHN (range: 1–25 years, one did not disclose). Interviews were co-facilitated by KL and AD in the first four instances, and the remainder were facilitated by KL. Interviews had a mean duration of 45 min (range: 21–85 min) and were conducted in a setting nominated by the participant.

### Analysis

Interviews were recorded using a digital recording device, transcribed using NVivo Transcription [39], and checked to ensure accuracy. Following interviews, KL and AD held debriefing meetings to discuss the experiences and readiness of each participant. The experiences of participants were also discussed and explored with the broader research team during weekly team meetings across the data collection period. Four interviews were selected and analysed by KL using line-by-line coding to develop an initial coding framework. These four interviews were selected as they illustrated a diverse range of participant readiness which enabled the development of a well-rounded coding framework. Constructed memos were also used in identifying key concepts, events and relationships between concepts. Next, the research team met to review the initial framework, key concepts, events and relationships and developed a coding structure for analysing all twenty interviews. During analysis, the research team met weekly to review the categories and sub-categories using constant comparison analytic process. Constant comparison allowed the research team to establish analytic distinction in the data which evolved throughout data collection and analysis [30]. Once transcripts had been coded, diagramming was used as part of the analytic process to explore the connections between categories [30, 40]. Application of case examples that demonstrated differing levels of participant readiness were used to test the rigour and completeness of the constructed theory and saw interactive refinements to the theory over numerous research team meetings [30, 40].

### Results

#### The Readiness of Doctors and Nurses to Assess and Address Patients’ Social Needs: Overview of the Theory

**Fig. 1:**
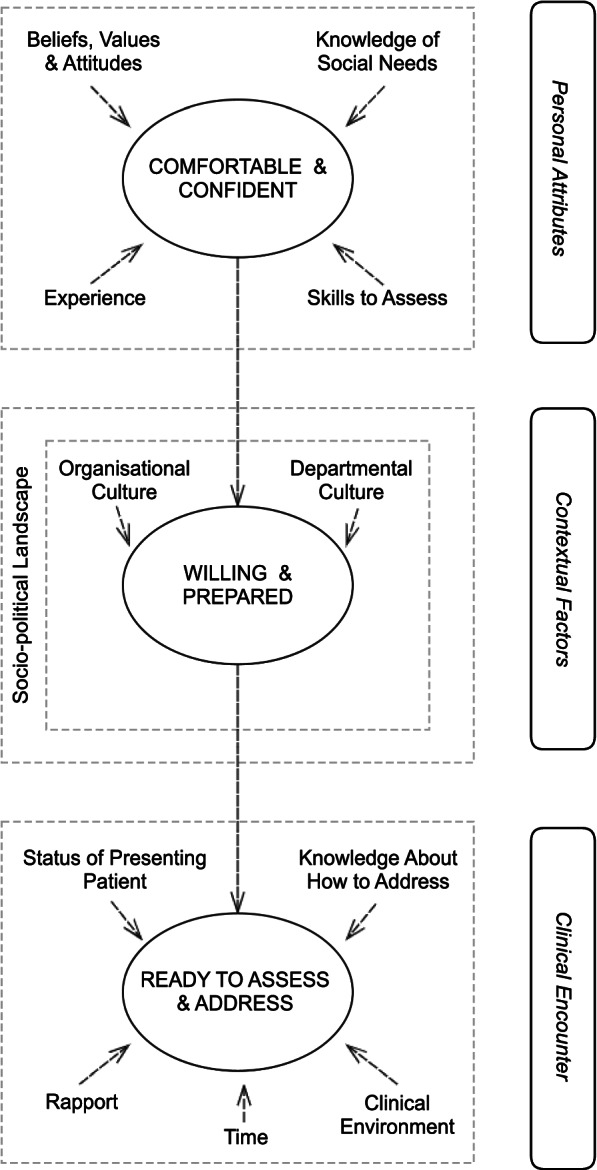
The Readiness of doctors and nurses to assess and address patients’ social needs

The constructed theory is visually depicted in Fig. 1. It demonstrates that while a clinician may be comfortable and confident to assess social needs, their readiness to address social need is influenced by the context in which they work and the nature of each clinical encounter. The readiness of senior doctors and nurses is founded upon a state of being comfortable and confident to assess social need as determined by a range of personal attributes (i.e., beliefs, values and attitudes, knowledge of social need; skills to assess social need, and experience); a state of being willing and prepared to assess and address social need facilitated by a supportive contextual environment (i.e., socio-political landscape, organisational and departmental culture); and enabling characteristics of the specific clinical encounter (i.e., status of the presenting patient, knowledge about how to address, time, clinical environment, and rapport) that facilitate a state of readiness to assess and address social need*.* We found readiness varies across individuals and clinical departments. The theory affords a fluidity to the readiness of doctors and nurses to assess and address social need on the basis of context, in that doctors and nurses may demonstrate readiness in one clinical context but not another, or also within one clinical encounter but not the next.

#### Personal attributes

Participants spoke of a number of personal attributes that influenced whether they felt comfortable and confident to assess and address patients’ social needs in the hospital setting. These included personal and clinical beliefs, values and attitudes; knowledge and understanding of social need; skills to assess social need; and previous personal and clinical experience.

#### Beliefs, values and attitudes

Doctors’ and nurses’ personal beliefs, values and attitudes appeared to intrinsically motivate actions to intervene on social factors: *“I think people who are attracted to it here, probably are attracted to work here because I feel that it’s an even greater area of need.” (Participant 2).* Clinical beliefs, values and attitudes appeared to provide a lens through which doctors and nurses viewed their role. These are shaped by training, previous and current clinical experiences, and the overall culture of their clinical specialty. They provided another aspect of intrinsic motivation for doctors and nurses, particularly in relation to whether assessing and addressing patients’ social needs in the hospital setting was within their role and/or responsibility: *“I find it as part of my role. Yeah, in terms of holistic care for [the patient]. And so, I don’t put it in this separate compartment. This is medical. This is social.” (Participant 13).* One senior clinician reflected on the differing intrinsic motivations of doctors and nurses in relation to assessing and addressing patients’ social needs:

*“They’re required to do it as part of their training process, but do they either have or want, which is a different concept, that skill set is different. Do they really regard it as important for them? I think the answer is not every one of them.” (Participant 10).*

Divergent perspectives were shared regarding the limits of the role of doctors and nurses. Some believed that it was the clinical role of doctors and nurses to be assessing and addressing patients’ social needs as part of healthcare provision: *“I think that we are nurses and midwives, and doctors need to instead of allocating or referring off to social work, we need all to be more informed about what are those resources” (Participant 4).* Others believed that it is not the role of doctors or nurses to assess and address patients’ social needs: *“Sometimes you just flag it as a social worker needs to go in and deal with it.” (Participant 1).*

#### Knowledge of social needs

Doctors and nurses demonstrated a high level of knowledge of patients’ social needs and the ways in which social factors impact on patient’s health in the NALHN catchment area. Social needs commonly identified included low levels of health literacy, access to and cost of transportation, social isolation, and inability to afford medication and treatment costs. As one participant identified, *“we move a service [from another site] to here we find we lose a percentage of patients because they can’t travel… to get here.” (Participant 12).* Doctors and nurses demonstrated that they used their knowledge to inform their clinical decision making in attempts to address the effects of unmet social needs on patient’s health outcomes: *“There’s no point sending them home with medications if they’re not going to fill the script because they don’t have the money.” (Participant 14).* Doctors’ and nurses’ knowledge of patients’ social needs also helped shape their attitudes and their ability to empathise with their patient population, as one nurse described: *“So just knowing, knowing why people might make decisions and things and being able not to judge, rather look at it from more of an objective point of view.” (Participant 14).*

#### Skills to assess

Assessing patients’ social needs was often done in the form of ‘social history taking’ as part of a routine medical assessment, something that had been embedded from the early stages of medical and nursing training. The skills of doctors and nurses to conduct an effective social history significantly contributed to their comfort and confidence to assess. Doctors and nurses relied on prior clinical training and experience to guide what questions they asked patients rather than using a formal social risk screening tool:

*“So, it’s not like they use a checklist of social determinants, but you know, it’s the same as if you’re taking in a history of somebody who comes in with a heart attack. You don’t have like a checklist of heart attack. You have a body of knowledge that you apply to taking a history from that patient.” (Participant 5).*

While undertaking a social history assessment was routine across all departments, the depth of questioning varied according to department, clinical role and the patient presentation, as one participant highlighted: *“…there’s definitely some form of social history taking for all patients. Yeah, it’s a matter of how much you delve into it.” (Participant 16).*

Some participants explored the use of structured screening tools as a way to support the readiness of doctors and nurses to assess patients’ social needs: *“I think trying to get the information in a planned way is important” (Participant 6).* However, many participants did not see screening tools as a silver bullet solution to assessing patients’ unmet social needs in a hospital setting, as one participant described: *“I think it would help gain the information. I don’t think it’ll make them any more comfortable asking the questions.” (Participant 16).*

#### Experience

Experience emerged as another key personal attribute associated with doctors’ and nurses’ comfort and confidence in assessing patients’ social needs in the hospital setting. While formal education and training were cited as important factors, doctors and nurses identified that a significant proportion of their knowledge and skills were a result of cumulative clinical experience: *“Well, it’s just interesting when you said has anyone actually taught you, and I was like well not specifically I don’t think anyone’s ever.” (Participant 19).* The value of clinical experience was highlighted by one senior doctor: *“Look, I think that I can capture what I need to capture for the needs of the person but that’s me maybe a junior doctor might feel differently.” (Participant 2)*. Past experience assessing and addressing patients’ social needs was a key determinant in doctors’ and nurses’ comfort and confidence: *“I’m comfortable with it because I’ve been doing it a long time.” (Participant 17).* While comfort and confidence differed across individuals these states were generally viewed as a natural progression for doctors and nurses determined by clinical experience.

Importantly, past negative experiences greatly impacted how comfortable some doctors and nurses were to assess and address social needs in the future. One interviewee described feeling distressed as a result of being unable to assist a patient in need due to a lack of available resources: *“And we just left it. And I feel awful, and I’m still thinking about it.” (Participant 1).* This impacted the doctor’s comfort and confidence to assess patients in the future: *“I’m worried that I might be trapped in a situation… where it might fall on me to try and sort out problems.” (Participant 1).*

#### Contextual factors

Participants provided numerous examples of contextual factors that impacted their readiness to assess and address social needs. Supportive contextual factors enabled doctors and nurses who were comfortable and confident to transition to a state of being willing and prepared to assess and intervene.

Willingness refers to the desire and motivation of a doctor or nurse to assess and address a patients’ social needs, influenced by personal and clinical beliefs, values and attitudes in the context of organisational culture and departmental culture. Preparedness refers to the skills, training, and knowledge of a doctor or nurse to be able to execute an assessment in the context of organisational culture and departmental culture. Both distal (i.e., social-political landscape) and proximal (i.e., organisational, and departmental culture) contextual factors influence the willingness and preparedness of doctors and nurses to assess and address social needs in the hospital setting.

#### Socio-political landscape

A number of participants drew attention to socio-political factors such as the resource allocation for the NALHN catchment area, including the hospital system and primary services such as GPs and community services. The local political context was also highlighted by doctors and nurses as impacting on NALHN health resourcing. As a region with secure political representation (the electoral seat in which NALHN operates is considered a ‘safe seat’ for the Australian Labor Party), some doctors and nurses felt that the government inequitably funded the hospitals and general infrastructure compared with other regions.

*“Look what’s happening down around [a different health network] these days. There is a truckload of money being poured into that area because it’s a swing vote area. This is not a swing vote area. And so, they just don’t get the resourcing by the government to be able to do things.” (Participant 10).*

Some recognised that there were barriers to patients accessing primary care, which resulted in hospital presentations:

*“So a lot of the GPs are overworked, a lot of them practice six-to-eight-minute consults. And if a patient goes there with two problems… they quite literally told which is the big one you want to talk about today, because we’ll have to talk about that one another day,” (Participant 10).*

Doctors and nurses also expressed frustrations with community services and organisations, noting that they were difficult to access which meant they struggled to assist patients in navigating their way to the additional supports and services they require.

*“People have nowhere else to go… they’re booked out for the next 12 months [or] they’ve closed their books… we have no stepdown services… all of the services that are available in the CBD [central business district] are not available in NALHN.” (Participant 11).*

The lack of primary and community-based support and resources was reported by doctors and nurses as placing pressure on the tertiary healthcare system which becomes a destination for the patients who have not been effectively managed in the community. Doctors and nurses perceived that the hospitals were bearing the brunt of social needs not being assessed and addressed in the primary and community sectors.

*“I think our role is very much while we are not a primary health care service, we are lobbying very hard because we have to stem the flow to tertiary, and at the moment all the flow is coming into tertiary because people have no other options.” (Participant 11).*

#### Organisational culture

The hospital culture is another factor influencing the willingness and preparedness of doctors and nurses. Organisational culture describes the values, behaviours and expectations within the hospital that influence the norms of the clinical setting and doctors’ and nurses’ clinical beliefs, values and attitudes. As one participant noted on the demands on doctors and nurses:

*“…and I guess health, you know such fast pace, but trying to admit patients, treat them and discharge them as quick as we can. And I think sometimes we maybe forget there’s a person you know, that might have other things that we need to consider.” (Participant 8).*

Organisational priorities and values also shaped doctors’ and nurses’ beliefs about the role of the hospital in assessing and addressing patients’ social needs: *“This is a tertiary hospital. So, we’re about dealing with tertiary problems, prevention should be out in the community.” (Participant 3).*

#### Departmental culture

The collective values, ideas and priorities within the clinical department, which we considered to be the departmental culture, was a determining factor in the perceived role and responsibility of doctors and nurses to assess and address patients’ social needs. The departmental culture appeared to influence whether doctors’ and nurses’ perceived assessing and addressing patients’ social needs to have direct relevance to their immediate clinical practice and a patients’ immediate clinical outcomes. Doctors and nurses in some departments believed assessing patients’ social needs was inherent to their clinical practice and that they would not be able to do their jobs without performing an assessment:

*“I think that as health professionals and whenever we see a patient that the social circumstances always come into it. So, I don’t think there’s any circumstance that we’re really where that doesn’t come into it.” (Participant 2).*

However, doctors and nurses from other departments and clinical disciplines perceived that assessing and addressing social need was outside their role. This led them to be unwilling and unprepared to assess and address patients’ social needs:

*“Well, I don’t know, it’s hard to answer, as I said, you know, if I go and see a doctor, I don’t want to be quizzed about things I haven’t been there to see them about.” (Participant 3).*

Often this was due to the clinical objectives of the department. Doctors and nurses across clinical disciplines gave diverging statements regarding whether they assessed, how they assessed, what they assessed and why they assessed social needs. Drivers of departmental culture (collective values, ideas and priorities) influenced whether doctors and nurses believed assessing and addressing was within their clinical role and responsibility. These included key performance indicators (KPIs), department role within the tertiary setting, the context of the social need, and clinical specialisation. For example:

*“We have to set up the social situation, because if we don’t set it up pre-surgery or pre-admission, then it becomes a problem to solve during their admission, which then prolongs their length of stay.” (Participant 12)*

#### Clinical encounter

We propose that the readiness of doctors and nurses to assess and address patients’ social needs in the hospital setting is founded upon personal attributes*,* influenced by contextual factors and is also dependent on the situational factors of each clinical encounter. Situational factors include the status of the presenting patient, knowledge of how to address social needs, available time, the nature of the clinical environment, and rapport. We propose it is possible that doctors and nurses could be both comfortable and confident, willing and prepared, but in the absence of enabling situational factors surrounding the clinical encounter, they may not be ready and able to assess and address patients’ social needs*.*

#### Status of presenting patient

Participants reported that the circumstances of a patient’s presentation influenced whether they assessed and addressed social needs. The decision to assess and address a patients’ social needs was dependent on the perceived severity of the patients’ needs: *“…some things are more important than others.” (Participant 2).* Incidences of domestic violence, homelessness or child protection were highlighted as high-risk social needs and were allocated a higher priority compared to other needs such as food insecurity or insufficient finances; this was often due to mandatory reporting requirements and a need for triaging scarce social worker resources.

#### Knowledge about how to address

Participants shared examples of barriers to addressing identified social needs, such as a lack of available services and uncertainty around referral pathways: *“I think on the whole that our teams do a pretty good job, where the barrier for us is having options to refer people to.” (Participant 11).* In some circumstances, this left doctors and nurses in situations where they did not know how to address significant unmet social needs, as a participant explained: *“…but I don’t know what to do with that and I just sort of sat there with her and I was like, I can’t problem solve this, and I don’t know what to do with it.” (Participant 1).*

One of the primary barriers to being ready to assess patients’ social needs was not knowing what to do with the information after conducting an assessment.

*“I think I probably more have an issue with it because I’m like, oh do you have enough money to get home? They’re like, no. And then I’m like, OK, well, what can I actually do about it? Like, OK, you opened the can of worms, you kind of want to be able to do something to help.” (Participant 14).*

Some doctors and nurses avoided assessing patients’ social needs due to a lack of knowledge of referral pathways and a shortage of tangible supports: *“I feel like maybe some people don’t ask the question because then they’re stuck.” (Participant 14).* This was attributed in part to the nature of medical training: *“Once you’ve highlighted that problem. Um, yeah, I don’t think we’re taught to the sort of follow up to it.” (Participant 7).* As a result, doctors’ and nurses’ expressed futility in their ability to address an identified social need: *“the amount of difference you can make is limited.” (Participant 13).*

#### Time

The presence of sufficient time to assess and address patients’ social needs was another factor impacting the readiness of doctors and nurses and was often determined by the clinical role of the department and influenced by distal contextual factors such as available resourcing. Participants identified that having enough time to complete an assessment and/or address an identified social need was a primary barrier to readiness: *“Barriers are that we haven’t got time, there’s always the next patient waiting.” (Participant 15).* Participants drew attention to the impact that a lack of time had on their ability to have a meaningful conversation with their patients about their social needs, as one doctor highlighted: *“…sometimes people are not going to disclose all of their needs if you don’t spend the time they require.” (Participant 2).* Participants appeared conscious of the fact that they needed to spend time in asking sensitive questions about patient’s social needs, with one nurse commenting: *“I honestly think that sometimes the nurses are just so busy they can’t sit down and it’s not a conversation you want to have fleetingly.” (Participant 14).*

#### Clinical environment

The environment for assessment, which brings with it other barriers and enablers to assessment such as time and privacy, was a factor in whether doctors and nurses would conduct an assessment. Participants noted that their ability to assess and address social needs was dependent on the clinical setting: *“In the acute setting, if you hit a red flag, the social worker comes in and that’s about it. In the outpatient setting there’s a bit more space to deal.” (Participant 1).*

#### Rapport

Rapport with a patient and the importance of the patient-clinician relationship was identified as a determining factor in conducting an effective assessment, as described:

*“And you do have to have a rapport with people to a degree if there are some significant social issues, because people aren’t just going to open up, or some people just, you know, it’s about them feeling… the trust.” (Participant 17).*

However, rapport with patients was often affected by time and clinical setting which had the potential to leave patients uncomfortable or disengaged, as one doctor explained:

*“I see how it plays out, if it’s when they sort of shut down a bit then your kind of like well, I don’t want to lose a relationship with you because I’m asking kind of more personal questions.” (Participant 1).*

## Discussion

We found that the readiness of doctors and nurses is dynamic and is impacted by a complex interplay of personal attributes along with contextual and situational factors. Doctors and nurses across some departments and clinical specialties see assessing and addressing social needs as core to their clinical role and responsibilities and within their capabilities, while others do not. Beyond the necessary personal attributes that led to a state of comfort and confidence to assess social needs, features of the working environment such as supportive departmental culture and adequate time were seen to promote willingness, preparedness and readiness (or, when in deficit, created barriers to these states).

This study contributes a nuanced understanding of the readiness of doctors and nurses to assess and address patients’ social needs in the hospital setting that extends previous conceptualisations of readiness in healthcare settings. In a study of the readiness of family doctors to respond to intimate partner violence, Po-Yan Leung (41 p.521) [41] defined readiness as “a psychological state that indicates the extent to which an individual is cognitively, motivationally, and emotionally inclined to embrace and respond to a phenomenon”. This definition considers the personal attributes of doctors and how they impact their readiness to identify and respond to intimate partner violence in the primary healthcare setting. However, it does not explore the contextual and situational factors identified in our study that influence the readiness of doctors and nurses. Based on our findings, we propose that the readiness of senior doctors and nurses to assess and address patients’ social needs in the hospital setting could be defined as dynamic and founded upon a state of being comfortable and confident to assess social need as determined by a range of personal attributes; a state of being willing and prepared to assess and address social need influenced by the contextual environment; and impacted by characteristics of each clinical encounter.

Our proposed theory of readiness considers both person-specific and context-specific factors in the hospital setting. These factors represent the enablers, barriers and determinants of clinical practice and will influence the implementation and uptake of any potential social needs interventions in this setting [42]. To ensure that hospitals can effectively meet the needs of their patients, we believe our proposed theory of readiness is integral to any efforts to design and implement social needs interventions. Atkins and colleagues [43] highlight that when implementing new practices, change is required in both individuals and systems and “changing behaviour requires an understanding of the influences on behaviour in the context in which they occur.” (43 p.2).

Our theory provides potential targets for interventions to strengthen the readiness of doctors and nurses to assess and address patients’ social needs in the hospital setting. By prioritising social needs interventions, hospitals can create supportive environments for doctors and nurses to undertake assessments by mitigating barriers during clinical encounters and equipping doctors and nurses with the skills to effectively direct patients to the necessary resources and supports [9]. As we have highlighted, the organisational and departmental context were important determining factors in the readiness of senior doctors and nurses to assess and address patients’ social needs in the hospital setting. From a health systems perspective, intervening on social needs will need to be a strategic imperative defined by hospital Boards and administrators in order to influence organisational culture and thereby promote the readiness of doctors and nurses.

When considering intervention options, the dimensions of readiness in our proposed theory (personal attributes, contextual factors, and the characteristics of the clinical encounter) provide potential intervention points to promote the readiness of doctors and nurses to assess and address patients’ social needs in the hospital setting. The identified personal attributes provide important considerations for the educational system responsible for the training of doctors and nurses. Consistent with our findings, Quiñones-Rivera and colleagues drew attention to a lack of provider knowledge to be able to address patients’ social needs [19]. Targeted curricula for trainees and continuing professional development programs for qualified doctors and nurses could improve their comfort and confidence to conduct an assessment. Likewise, training programs centred around strengthening the capacity of doctors and nurses to identify and navigate referral pathways and support services could promote readiness to address identified social needs.

Some participants in this study noted that after identifying patients’ unmet social needs their preferred course of action was to refer to the social work department. However, it was also apparent from participants that there was a lack of available referral pathways and under resourcing of social work and community services. An increased investment in specialised social support roles and social work personnel in hospitals could promote the readiness of doctors and nurses to identify areas of unmet social needs and refer onto personnel who are adequately resourced to intervene. Bibbins-Domingo [44] suggests that professional teams including social workers, discharge liaisons nurses, community service liaisons, allied health professionals in conjunction with doctors and nurses can work as a multi-dimensional team to improve the outcomes for patients in the hospital and in the community. Maani & Galea [45] have also noted that there is a need to increase the number of staff whose core responsibility is focusing on population health interventions and whose skills are specific to those responsibilities. However, developing a social care workforce in the hospital system challenges the historically held perceptions of the expectations and responsibilities of doctors and nurses [46]. Further research, for example, could investigate provider decision making informed by readiness as well as the optimal mix of allied health and social work staff alongside medical and nursing personnel necessary to effectively assess and address patients’ social needs.

With a greater understanding of what constitutes the readiness of doctors and nurses in this context, hospitals can actively support their workforce to intervene on patients’ social needs [44]. Given that the readiness of doctors and nurses is a state highly influenced by contextual factors, any efforts to strengthen readiness would need to consider organisational-level strategies that target the working environment. Our theory of the readiness of doctors and nurses could be used to inform hospital quality improvement initiatives that consider working environments in addition to workforce capacity building. Other research has considered the utility of hospitals as a site for population health initiatives. Ramiah [9] suggests that understanding the capabilities of the healthcare workforce can assist organisations in setting internal goals, provide direction for long-term objectives and aid in the allocation of resources.

In hospitals that prioritise social needs interventions, an understanding of the readiness of doctors and nurses to address social needs can be considered in conjunction with behaviour change theory and implementation science to design targeted interventions and effective implementation approaches. The Theoretical Domains Framework (TDF) is just one of many models that could be applied to develop theory-informed intervention strategies [43]. The TDF represents a synthesis of 33 theories of behaviour change into 14 domains that consider both person-specific (e.g. social/professional role and identity, motivation and goals) and context-specific factors (e.g. environmental context and resources) [43]. Once a targeted intervention has been developed, models such as the Consolidated Framework for Implementation Research (CFIR) can be applied to identify any potential barriers and enablers to the implementation of the intervention [47]. There are numerous examples of implementation efforts that have applied behaviour change theory and implementation frameworks such as the TDF and CFIR to strengthen the effectiveness of interventions to change clinical practice [48–50]. While hospitals do have a key role to play in improving healthcare delivery to at risk populations, they are also at the behest of funding and resource allocation from government, as highlighted by a number of participants in this study. The primary healthcare sector is challenged, which increases the burden on the tertiary system. A systems perspective warrants consideration when contemplating ways in which to improve health services for communities experiencing disproportionate social and health inequalities. Understanding the readiness of doctors and nurses and the inclusion of social care in hospitals opens a broader discussion about the role and responsibility of hospitals as organisations in addressing social inequalities and the burden of disease in communities. This study contributes evidence necessary for governments and policy makers to reconsider adequate resourcing of healthcare systems so that they can better meet the needs of their communities. While hospitals should be considered part of a system-wide approach to reducing the social inequalities that lead to adverse health outcomes, Marmot & Allen [51], Braveman [52] and Andermann [53] argue that to mitigate the effects of social disadvantage on health outcomes, upstream SDH also need to be addressed by community leaders and policy makers. Taking action on housing, education, employment, community environments and access to primary healthcare from a structural level can improve individual’s SDH and thus reduce the burden of disease attributed to social factors [52].

### Strength and limitations

A strength of this study was the rigour applied to the development of the constructed theory. The research team included five researchers, each bringing a particular lens to the interpretation of data (i.e., clinical experience including both medical and allied health specialties, and academic experiences including clinical and population health perspectives) which reduced the risk that any singular biases could dominate the analysis. A limitation of this study was the targeted recruitment of participants from the same organisational setting which may impact the generalisability of findings to other settings. It is also possible that the senior doctors and nurses who agreed to participate in this study held stronger views than other staff. However, we specifically recruited experienced doctors and nurses who held senior roles (including administration) to capture the perceptions of staff who frequently set the departmental tone. We intend to explore the readiness of less experienced medical and nursing staff and allied health professionals in future work. There is also potential to examine whether hospital-based doctors and nurses in other settings hold similar views on readiness, and to extend this work to explore readiness in the primary care setting.

## Conclusion

The readiness of doctors and nurses to assess and address patients’ social needs in a hospital setting is founded upon a combination of personal attributes and influenced by contextual factors and a confluence of factors relating to each clinical encounter. This research has highlighted the divergent readiness of senior doctors and nurses and the interplay between doctors’ and nurses’ personal attributes and both distal and proximal contextual factors. If hospitals are to consider tailoring service delivery to incorporate interventions to assess and address SDH and social needs, they must consider strategies aimed at strengthening both doctors’ and nurses’ capabilities as well as the enabling characteristics of their working environments. They should also consider bolstering social health and allied health workforce across hospital departments. 

## Data Availability

The datasets used and/analysed during the current study are not publicly available due to privacy and confidentiality agreements but are available from the corresponding author on reasonable request.

## Abbreviations

SDH: Social determinants of health
HCW: Healthcare workers
NALHN: Northern Adelaide Local Health Network
KPIs: Key performance indicators
CBD: Central Business District
TDF: Theoretical Domains Framework
CFIR: Consolidated Framework for Implementation Research
