# Correction to: Exploring the readiness of senior doctors and nurses to assess and address patients’ social needs in the hospital setting

**DOI:** 10.1186/s12913-022-07758-0

**Published:** 2022-03-18

**Authors:** Katherine J. Lake, Mark A. Boyd, Lisa Smithers, Natasha J. Howard, Anna P. Dawson

**Affiliations:** 1 School of Public Health, The University of Adelaide, Adelaide, South 5005 Australia; 2 Adelaide Medical School, Faculty of Health and Medical Sciences, The University of Adelaide, Adelaide, South Australia 5005 Australia; 3 Northern Adelaide Local Health Network, Adelaide, South Australia 5000 Australia; 4 School of Health and Society, The University of Wollongong, Wollongong, New South Wales 2522 Australia; 5 Wardliparingga Aboriginal Health Equity, South Australian Health and Medical Research Institute, Adelaide, South 5000 Australia

**Correction to: BMC Health Serv Res 22, 246 (2022)**

**https://doi.org/10.1186/s12913-022-07642-x**

Following publication of the original article [1], the authors identified an error in the author affiliations’ list. Author affiliations 3 and 5 should be exchanged, and the author affiliation 2 needs to be updated.

The author affiliations have been updated in this correction article and the original article [1] has been corrected.
